# Liquid metal enabled conformal electronics

**DOI:** 10.3389/fbioe.2023.1118812

**Published:** 2023-02-03

**Authors:** Bingyi Ping, Guanxi Zhou, Zihang Zhang, Rui Guo

**Affiliations:** Department of Biomedical Engineering, Tianjin University, Tianjin, China

**Keywords:** liquid metal, conformal electronics, electronic skin, adhesive electrodes, bioelectronics

## Abstract

The application of three-dimensional common electronics that can be directly pasted on arbitrary surfaces in the fields of human health monitoring, intelligent robots and wearable electronic devices has aroused people’s interest, especially in achieving stable adhesion of electronic devices on biological dynamic three-dimensional interfaces and high-quality signal acquisition. In recent years, liquid metal (LM) materials have been widely used in the manufacture of flexible sensors and wearable electronic devices because of their excellent tensile properties and electrical conductivity at room temperature. In addition, LM has good biocompatibility and can be used in a variety of biomedical applications. Here, the recent development of LM flexible electronic printing methods for the fabrication of three-dimensional conformal electronic devices on the surface of human tissue is discussed. These printing methods attach LM to the deformable substrate in the form of bulk or micro-nano particles, so that electronic devices can adapt to the deformation of human tissue and other three-dimensional surfaces, and maintain stable electrical properties. Representative examples of applications such as self-healing devices, degradable devices, flexible hybrid electronic devices, variable stiffness devices and multi-layer large area circuits are reviewed. The current challenges and prospects for further development are also discussed.

## 1 Introduction

Conventional electronic devices used for biomedical applications normally are manufactured by printing circuits on a rigid two-dimensional surface. So far, some printing and assembly methods which are based on rigid conductive materials with special structures have been developed. These methods give the two-dimensional surface circuits better compliance and stretchability. The methods have been applied in biomedical detection (Kim et al., 2016; Ma et al., 2020). However, most of the application of flexible electronics are sophisticated and deformable three-dimensional surfaces. For example, human skin is a soft three-dimensional surface which has complex micro-fold structure. Therefore, these two-dimensional planar circuits cannot completely fit with the micro-fold structure of the skin. It is difficult to apply them stably on the human skin for long-term monitoring of physiological signals on the body surface (Ferrari et al., 2018). The connection between these devices and the electrode-tissue interface has stiffness and plane features, hindering the ability of these devices to sample and stimulate tissues (Polikov et al., 2005). Therefore, conformal electronic devices which can be stably adhered to random three-dimensional surfaces have gradually aroused people’s interest in researching (Huang et al., 2019). At present, numerous new conductive materials and their fabrication have been developed. These materials are applied in personalized healthcare, artificial implants, wearable electronic devices and other fields (Chu et al., 2016; Yang et al., 2018; Pan et al., 2020; Tringides et al., 2021).

Fabrication strategies for wearable and implantable three-dimensional conformal electronics typically fall into two categories. One using rigid metallic materials with higher conductivity and the other using conductive polymer materials with higher adhesion to human tissue and greater deformability. However, although rigid metal materials have excellent conductivity, there is an obvious mismatch between the mechanical properties of these materials and human tissues. The assembly strategy of some serpentine structures can make rigid metal wires obtain certain stretchability, but its manufacturing process significantly increases the cost of conformal electronics. It is not suitable for a wide range of applications (Wang Y. et al., 2018; Sim et al., 2019; Liu et al., 2022). Conductive polymers, hydrogels and other soft deformable materials have similar mechanical properties to human tissues. They are often used to manufacture conformal electronic devices that can be affixed to the surface of skin or organs (Zhang et al., 2020; Wang Y. et al., 2021; Xu Z. et al., 2021; Maithani et al., 2021). In addition, conductive hydrogels have high adhesion to human skin and organs. More importantly, these conductive polymers have excellent deformability and can be closely attached to the microfolds of the skin, which can be used for long-term electrocardiogram (ECG), electroencephalogram (EEG) and organ surface electrical signal monitoring (Kim et al., 2019; Lee et al., 2020; Kim et al., 2021; Wang C. et al., 2022; Sang et al., 2022; Tan et al., 2022). At the same time, by using the self-healing ability of hydrogel materials, a series of self-healing circuits have also been developed (Han et al., 2021; Tang M. et al., 2022). Some conductive polymers have good fluidity and processability and can be printed directly on the surface of human tissue by 3D printing (Muth et al., 2014; Zhu et al., 2018). However, due to their much lower electrical conductivity than rigid metals, conducting polymers and hydrogels are difficult to realize complex data processing and signal transmission. Recently, many conformal electronic devices based on silver paste and carbon-based materials have been developed. Generally, the silver paste and carbon based conductive materials are made to particles to form conductor. However, particle to particle is point contact which loses a little conductivity. Because of the accumulation of silver or carbon particles, these pastes can not be stretched without limitation. Besides, the silver is more expensive than gallium. It will cost more to transfer the silver into particles. Therefore, the development of three-dimensional conformal electronic devices with both high electrical conductivity and high deformation properties has become an important research direction.

Recently, room temperature LM based on low melting point gallium (melting point 29.8°C, boiling point 2204°C) has gradually attracted the attention of researchers because of their unique properties, such as excellent fluidity, high conductivity (3.4 × 10^6^ S·m^−1^) and good biosafety (Zhu et al., 2013; Kim et al., 2018; Markvicka et al., 2018). The gallium and its alloys, which combine the advantages of metal and liquid, are widely used in flexible sensors, intelligent robots, wearable electronics and other fields (Sheng et al., 2014; Zhang et al., 2015; Gao et al., 2017; Guo et al., 2018a). At present, many flexible electronic printing methods based on LM have been developed, such as microfluidic channels injection (Choi et al., 2017), spray printing (Guo et al., 2018c), mask printing (Guo et al., 2018b), direct writing (He et al., 2021), mechanical damage (Ren et al., 2020) and metal weighting (Li et al., 2015). They hold a promising potential for manufacturing conformal electronic devices on the surface of human skin. In addition, the biosafety makes LM be used as an implantable device for monitoring physiological signals in the body or treating diseases (Sun et al., 2017). However, the high surface tension and high fluidity of LM make it difficult to form circuits on curved surfaces stably. Therefore, it is necessary to summarize the research in recent years on strategies to improve the conformal ability of LM on three-dimensional surfaces.

**FIGURE 1:**
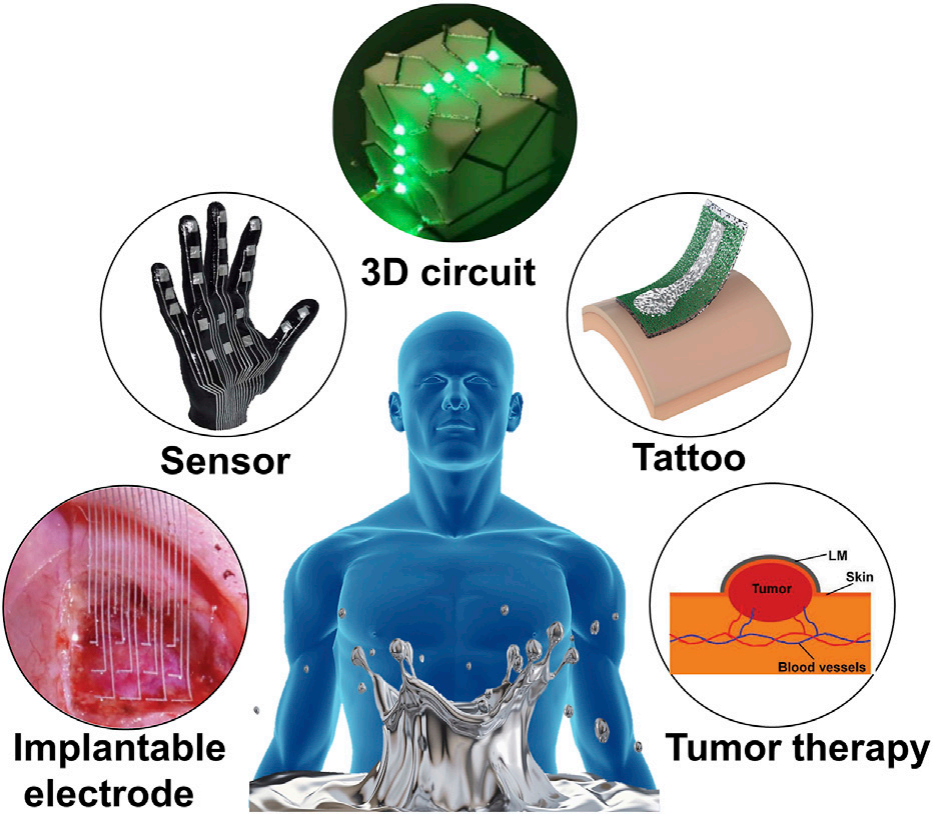
Various LM enabled conformal electronics.

In this review, we discuss various methods of printing the LM on three-dimensional surfaces, which are used for the fabrication of conformal electronic devices on the surface of human tissue. Initially, the unique properties of LM, including metal properties, liquid properties and biomedical safety, are discussed to demonstrate their advantages over other flexible conductive materials. After that, we summarize the methods of LM conformal electronic printing on deformable substrates based on different strategies. On these substrates, LM exists in the form of bulk metals or micro-nano droplets. LM can obtain conformal ability with human tissues while maintaining high conductivity. Moreover, these substrates have excellent adhesion to human tissues and can be used for long-term health monitoring. Finally, we discuss conformal electronic devices with different functions based on the special properties of LM and flexible substrates (Figure 1). The unsolved technical problems and potential future applications of common electronic devices based on LM are also discussed.

## 2 Unique properties of LM

LM refers to the metal or alloy whose melting point is near room temperature, such as mercury, gallium, cesium and perylene. However, mercury is volatile and highly toxic, while cesium and cesium are extremely active and radioactive. Once improperly operated or stored, it is easy to cause unimaginable harm to organisms and the surrounding environment, so it is difficult to use a large number of them in daily life. Therefore, gallium and gallium-based alloys with non-volatile, low toxicity and good biosafety have gradually become the focus of researchers. In addition to gallium metal, gallium-based alloy materials can also remain liquid at room temperature.Moreover, the melting point of the alloy can be adjusted by changing the type of metal. And for the same kind of alloy, the change of its mass ratio will also affect the melting point of the alloy. For example, eutectic gallium indium alloy (EGaIn_24.5_) and gallium indium tin alloy (Galinstan: Ga_68.5_In_21.5_Sn_10_) have a lower melting point than the elemental metal gallium and can be kept in a liquid state at room temperature. So alloy with low enough melting point can be chosen to avoid losing flexibility when working under low temperature. Although in winter, the human skin and the clothes can make enough warmth for LM circuit working. Gallium and its alloys have good electrical conductivity, thermal conductivity, good liquid fluidity, and its melting point near room temperature makes it easy to switch between solid and liquid forms, so it has more potential applications than traditional metals. In this chapter, the unique properties of some gallium-based LM conducive to the manufacture of three-dimensional conformal electronics for biomedical applications will be discussed. The physical properties of some gallium-based LM are summarized in Table 1 (Morley et al., 2008).

**TABLE 1:** The physical properties of gallium-based LM.

Composition	Ga	GaIn_24.5_	Ga_67_In_20.5_Sn_12.5_	Ga_61_In_25_Sn_13_Zn_1_
Conductivity (S m^−1^)	3.7×10^6^	3.4×10^6^	3.1×10^6^	2.8×10^6^
Melting point (ºC)	29.8	15.5	10.5	7.6
Boiling point (ºC)	2,204	2000	>1,300	>900
Density (kg/m^3^)	6,080	6,280	6,360	6,500
Viscosity (m^2^/s)	3.24 × 10^−7^	2.7 × 10^−7^	2.98 × 10^−7^	7.11 × 10^−8^
Surface tension (N/m)	0.7	0.624	0.533	0.5

## 3 Metal characteristics

**FIGURE 2:**
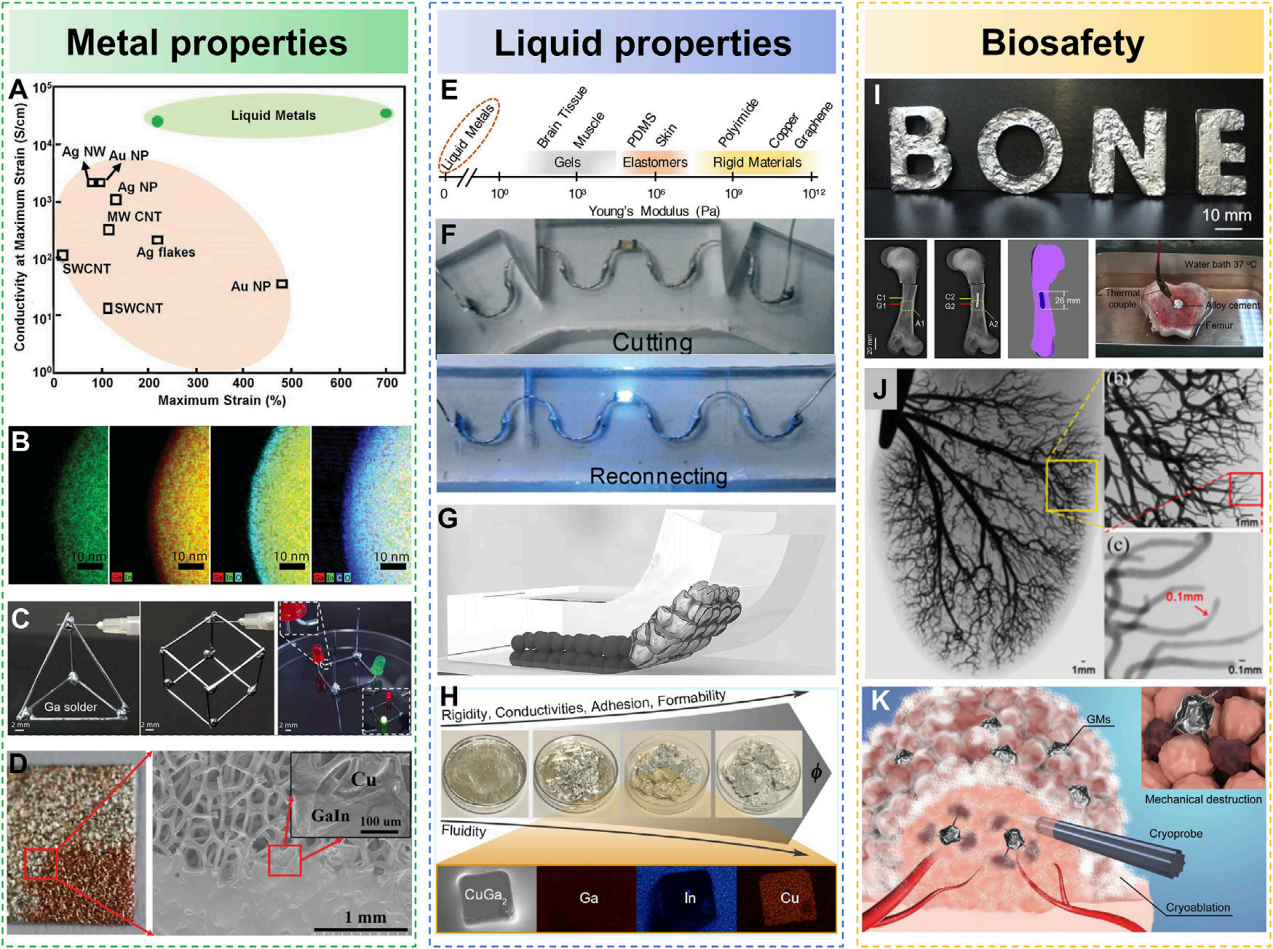
The unique properties of LM. **(A)** The conductivity of LM is compared with other common flexible conductive materials (Wang et al., 2018c). Copyright@2018, Wiley **(B)** Oxide film on the surface of LM (Lin et al., 2015). Copyright@2015, Wiley **(C)** The solid-liquid phase transition of LM is used for welding three-dimensional circuits (Wang et al., 2022b). Copyright@2022, Elsevier **(D)** Wetting of LM on the surface of porous copper (Ma et al., 2018). Copyright@2018, RSC **(E)** Young’s modulus of LM is compared to that of common flexible materials (Wang et al., 2018b). Copyright@ 2018, Wiley **(F)** Self healing of LM in micro channels (Li et al., 2016). Copyright@2016, RSC **(G)** Conductive composite based on LM micro nano particles (Tang et al., 2018). Copyright@2018, Cell Press **(H)** Semi LM with low fluidity (Tang et al., 2017). Copyright@ 2017, ACS **(I)** Bone cement 247 based on LM (Yi et al., 2014). Copyright@ 2014, Elsevier **(J)** LM is used for angiography (Wang et al., 2014). Copyright@ 2014, IEEE **(K)** LM microparticles phase change medicated mechanical destruction for enhanced tumor cryoablation (Sun et al., 2020a). Copyright@ 2020, Wiley.

**High conductivity:** Generally speaking, the electrical conductivity of metallic materials is much higher than that of non-metallic materials. Figure 2A shows the high conductivity of LM compared to other commonly used flexible conductive materials (Wang et al., 2018c). For example, the conductivity of EGaIn(3.4 × 10^6^ S·m^−1^), which is commonly used in the preparation of flexible circuits, is much higher than that of other non-metallic materials (Carbon: 1.8 × 10^3^ S·m^−1^, CNT: 5.03 × 10^3^ S·m^−1^, PEDOT:PSS: 8.25 × 10^3^ S·m^−1^).In addition, the adjustment of metal types and proportions in LM alloys and the different content of dopants will affect the conductivity of LM materials. Therefore, we can adjust the metal ratio of LM alloy or add different materials into it to change the conductivity of LM materials.

**Surface oxidation:** The surface tension of gallium-based LM at room temperature is in the range of 500–700 mN m^−1^. But because gallium exposed to air is easily oxidized by oxygen, an oxide film with a thickness of about 3 nm is formed on the surface of LM droplets (Lin et al., 2015). The main component of this oxide film is gallium oxide. Figure 2B shows the thickness and composition of the oxide on a particle through energy-dispersive X-ray spectroscopy (EDS) mapping using a transmission electron microscope (TEM). This solid oxide film can significantly reduce the surface tension of the LM, and make the LM easily adhere to the surface of other substrates. Because this oxide film is thin enough, it will not have obvious influence on the conductivity of LM. In addition, other kinds of oxides can be formed on the surface of LM, and alumina can be formed by adding other metals, such as metal aluminum (Lin et al., 2020).

**Solid–liquid phase transition:** Most metals have a high melting point, while the melting point of LM is close to room temperature. Therefore, the LM can respond to the change of external temperature at room temperature, causing it to switch between solid and liquid. This solid-liquid phase change can cause significant changes in thermal conductivity, hardness, shape and volume of LM, and can be used to manufacture three-dimensional circuits with adjustable stiffness. For example, Bhuyan et al. developed a room-temperature welding method to assemble metal wires of three-dimensional structure (Wang et al., 2022b), as shown in Figure 2C. This three-dimensional conductive structure is composed of solid metal gallium wires, and its joints are welded by LM gallium droplets. The liquid gallium is placed at the joint to wet with the solid gallium wire, and when it is restored to room temperature, the gallium droplet solidifies and is welded to the solid gallium wire. This three-dimensional structure assembled by independent metal gallium wires has excellent conductivity and can be used as a 3D circuit for lighting LED lamps. Even after gallium melts, the circuit can still keep conductivity.

**Metal weldability:** It is well known that metal welding is caused by the diffusion of atoms between metals. The common solid metal surface is attached to a very thin layer of air, blocking the process of metal welding. Therefore, the welding of solid metal with high melting point requires high temperature to melt the metal and make the two metals in close contact. LM with lower melting points do not need to be melted at high temperature, so they can be welded with solid metals at room temperature. However, a metal oxide film is easily formed on the surface of LM, which blocks the welding process between LM and solid metal. To solve this problem, He and others developed a reactive-wetting method based on electrochemistry to realize the coating and spreading of the LM on the porous Cu surface (Ma et al., 2018), as shown in Figure 2D. In this study, LM droplets are placed on porous copper plates, which are completely immersed in sodium hydroxide solution (0.05 mol/L). The cathode is connected to the copper plate and the anode is placed in the solution. We can see that the LM spreads rapidly on the surface of the copper plate and infiltrates into the porous structure of the copper plate by applying the direct current voltage of 10 V between the two electrodes. This method can be used to prepare large-area conductive CuGa2 films, realizing the transfer printing technique of flexible electronics.

### 3.1 Liquid characteristics

**Fluidity:** LM can maintain a liquid state with excellent fluidity at a temperature above the melting point. This fluidity enables LM printed on a flexible substrate to deform with the substrate without limiting the deformability of the substrate. Figure 2E shows the Young’s modulus of LM, compared to other common flexible materials (Wang C. et al., 2018). At present, numerous flexible circuit preparation methods have been developed based on the fluidity of LM. For example, photolithography is used to prepare polydimethylsiloxane (PDMS) microchannels with specific structures, and then the LM is injected into the microchannel to form an intrinsically stretchable conductor (Khoshmanesh et al., 2017).LM has infinite stretching property in theory, so the stretching property of LM flexible circuit fabricated by perfusion method depends on the mechanical properties of the polymer making flow channel. For example, by pouring LM into a silicone tube, a conductive fiber with an elongation of more than 1,000% can be produced (Wang et al., 2022c). This conductive fiber can be woven into clothing fibers and used to make wearable electronic devices. In addition to clothes, they can be directly printed on human skin to monitor heart rate, breath rate, or even blood pressure when combined with other conventional sensors. Besides, Majidi and others developed a technology of freezing LM for rapid fabrication of three-dimensional electronic devices with complex structures (Fassler and Majidi, 2013). In this study, the LM with high melting point is injected into the mold made of flexible material to fabricate liquid models and circuits with three dimensional structure.

**Self-repair:** Flexible materials are prone to structural damage in the process of long-term use or frequent deformation. Because this kind of damage and scratches are difficult to avoid, realizing the self-repairing ability of flexible electronics can greatly improve the stability of electronic equipment. At present, stretchable conductors with self-repairing function can be realized by pouring LM into polymer channels with self-repairing ability. When the polymer channel is cut off, the exposed LM contact with air is rapidly oxidized, and the metal oxide film formed on its surface can limit the flow of LM and prevent it from leaking out of the fracture of the channel. When the damaged interface is reconnected, the oxide film on the surface of the LM is extruded and ruptured, causing the LM to remerge (Li et al., 2016). Figure 2F shows the self-repairing ability of LM circuits.

**Size tunability:** LM is easily broken into tiny droplets under the function of external mechanical forces, such as high-pressure airflow, ultrasonic and mechanical stirring. For example, Jiang and others created a conductive ink based on LM droplets (Tang et al., 2018), as shown in Figure 2G. Firstly, the LM is added to an organic, volatile solvent (n-decyl alcohol). And under the function of ultrasound, the LM is broken into droplets with a diameter of 5 microns, whose surface is wrapped by n-decylalcohol. Because the LM droplets are separated from each other, in order to make the LM droplets form a conductive path, it is necessary to use external mechanical forces to destroy the droplets and make the separated LM droplets communicate with each other, and finally form a conductive path. This LM ink can be printed on a variety of substrates and maintain good electrical conductivity and stability in a high tensile state.

**Semi-LM mixture**: Some methods have been developed by doping solid metal particles into LM. Tang et al. immersed the LM in the sodium hydroxide solution, and then added solid metal copper particles to it. After that, under the stimulation of the external electric field, copper particles can infiltrate into the LM after a period of time to form a complex of copper and LM(Tang et al., 2017). As shown in Figure 2H, the fluidity of this composite is greatly reduced compared with that of LM, and its fluidity gradually decreases with the increase of the proportion of doped solid metal particles. In addition, the conductivity of the semi LM material is significantly improved compared with that of the LM, and the conductivity of the semi Cu-EGaIn with the copper powder doping ratio of 20% can be increased to 6 × 10^6^ S·m^−1^, much higher than EGaIn. (3.4 × 10^6^ S·m^−1^).

#### 3.2 Biosafety

Gallium based LM have good biosafety. Due to low vapor pressure and limited solubility in water, LM based on gallium can hardly enter the human body, and its cytotoxicity is far lower than that of highly toxic metal mercury (Wang et al., 2018c; Lin et al., 2020). For example, studies have shown that the melting point of bismuth-indium-tin-zinc alloy (Bi/In/Sn/Zn) based on bismuth can reach 57.5°C. It is a solid state in the body environment and requires only slight heat to melt into a liquid. In addition, this alloy material has high mechanical strength and can be used as bone cement to fill damaged bones to achieve the role of bone repair (Yi et al., 2014), as shown in Figure 2I. Furthermore, LM is a metal material with high density, so it has a strong blocking effect on x-rays. Combined with its flowable characteristics, it can be used as an angiography agent to improve the imaging ability of blood vessels under X-rays. And compared with traditional angiography agents, LM has a stronger imaging contrast. Previously, some studies injected metallic gallium into blood vessel rich organs such as isolated heart and kidney (Wang et al., 2014). It can be seen from Figure 2J that the LM poured into the organs fills the blood vessels, and the capillaries in the organs still have a good imaging effect in the CT images. This technology can realize the visualization of capillary distribution *in vitro*. In recent years, there has been a lot of research on breaking LM into micro nano particles, and modifying the surface of these LM micro nano particles with drugs, so that they can enter the human microenvironment and have a specific therapeutic effect on pathological biological tissues, such as tumor treatment (Yu and Miyako, 2017). For example, Lu et al. dispersed LM into micro droplets by ultrasound, and attached drugs to their surfaces using these micro droplets as carriers to prepare pH responsive micro drug loading robots (Lu et al., 2015). Changing the temperature can make LM micro nano particles transform between solid and liquid (Sun et al., 2020a; Sun et al., 2020b). In the phase change process from liquid to solid, the surface morphology of LM micro nano particles changes, and the generated spikes can destroy the surrounding tumor cells, thus achieving the effect of killing tumor cells, as shown in Figure 2K.

## 4 Preparation method

Numerous preparation methods have been developed in order to fabricate LM patterns on three-dimensional curved surfaces, especially on the surface of human tissue. According to the various principles and different circuit substrates, these preparation methods are divided into the following six types.

### 4.1 Direct printing on the skin

**FIGURE 3:**
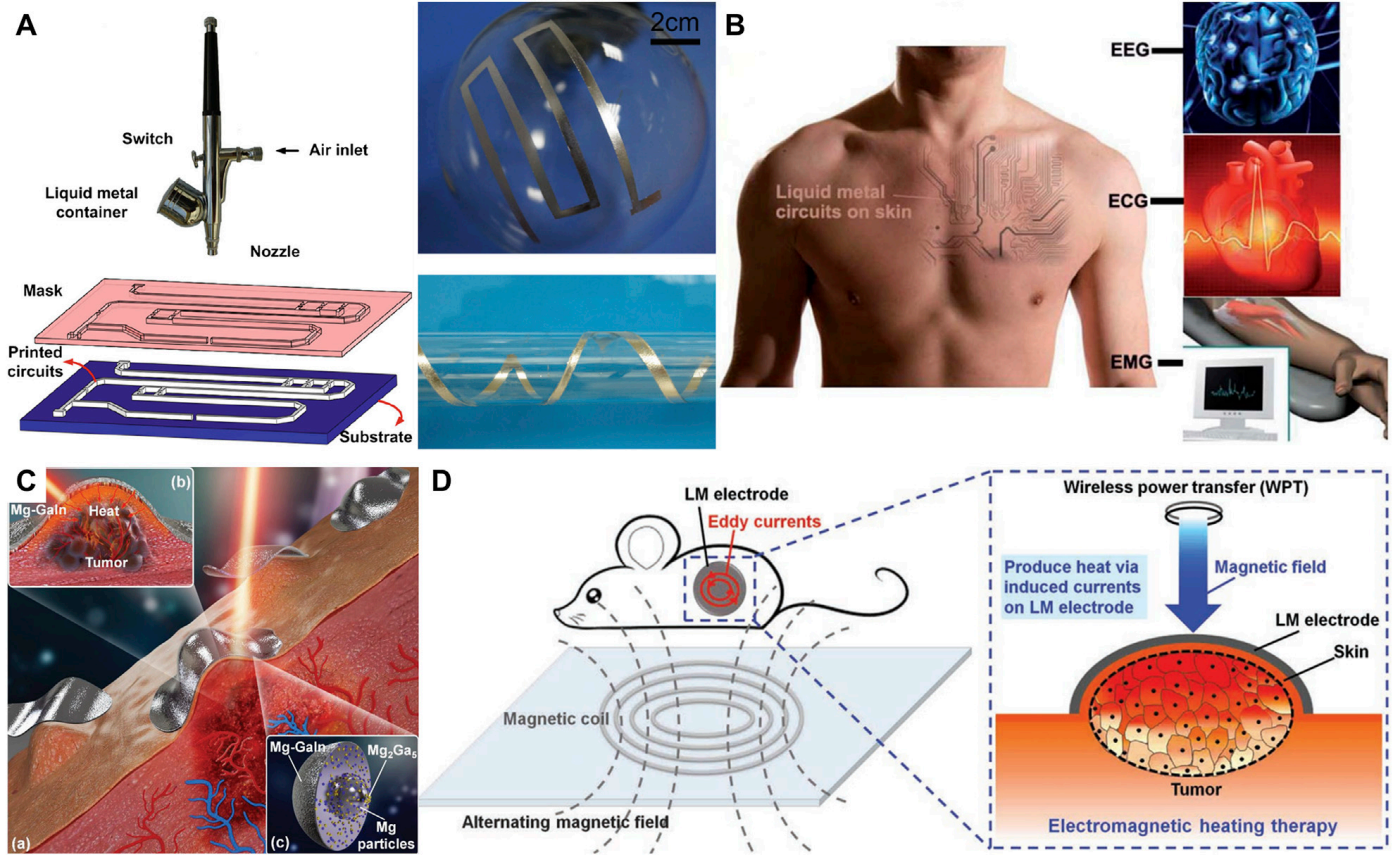
Methods of preparing LM circuits directly on the skin. **(A)** The schematic diagram of the rapid prototyping circuits based on atomized spraying of LM droplets (Zhang et al., 2014). Copyright@2014, Springer **(B)** Schematic conception of fabricating LM circuits on human skin (Guo et al., 2014). Copyright@2014, RSC **(C)** The soft Mg-GaIn mixtures applied on skin–tumor surface (Wang et al., 2018d). Copyright@2018, Wiley **(D)** Schematic illustration of conformable O-291 GaIn bioelectrodes on in vivo tumors (Wang et al., 2019). Copyright@2019, Wiley.

Due to the high surface tension of the LM, it is difficult to directly adhere the LM to the rough surface of the skin with many wrinkles. However, previous studies have shown that the surface oxide film formed by the oxidation of LM in the air can greatly reduce the surface tension of LM droplets. Besides, under the action of mechanical external forces, LM can be broken into tiny droplets to increase the surface oxide (Wang and Liu, 2015). Thus, Liu et al. have developed an atomized spraying method of LM droplets (Zhang et al., 2014). This method uses a high-pressure spray gun to break LM into micron-scale droplets that are sprayed directly onto the flexible substrate. Figure 3A shows the schematic diagram of the rapid prototyping circuits based on atomized spraying of LM droplets. The LM can be printed on paper, silica gel, plants, and even on the surface of three-dimensional objects. Particularly, based on the advantage that this spraying method can make LM circuits on rough substrates, Liu et al. have also successfully sprayed LM on the surface of the skin to make conformal circuits on the body surface, as shown in Figure 3B (Guo et al., 2014). The LM circuit made by this method can follow the deformation of the skin and can be used to monitor a variety of physiological electrical signals on the human body surface for a long time. However, the shortcomings of this method are also evident. Firstly, the operation is sophisticated, requiring specific spraying equipment and mask plate. Secondly, the spraying process will cause a waste of LM. In order to avoid the waste of LM caused by spraying steps, Liu et al. developed a laser heated Mg-GaIn material, which can be printed directly on the surface of the skin for tumor treatment, as shown in Figure 3C(Wang et al., 2018d). In this study, the material was prepared by mixing gallium-based LM with magnesium particles. This material shows great compliance, flexibility, formability, thermal conductivity, biocompatibility, photothermal efficiency, and excellent properties of ablation of tumors inside the body. Mg-GaIn materials can be applied directly to the skin to achieve minimally invasive thermotherapy of tumors without injection into the body. Except doping metal particles into LM, the LM can be thoroughly exposed to the air and oxidized by stirring the LM in the air. Then the oxidation gain mixed numerous oxides (O-GaIn) can be obtained (Wang et al., 2019). This material has evidently increased adhesion to the skin and is easier to form, especially as a conformal bioelectrode to cover abnormal tumors. Figure 3D shows that the O-GaIn electrode is applied to the surface of the mouse subcutaneous tumor. The electromagnetic thermal effect produced by the LM electrode is used to heat the tumor, successfully preventing the growth of the mouse tumor.

### 4.2 Mold printing

**FIGURE 4:**
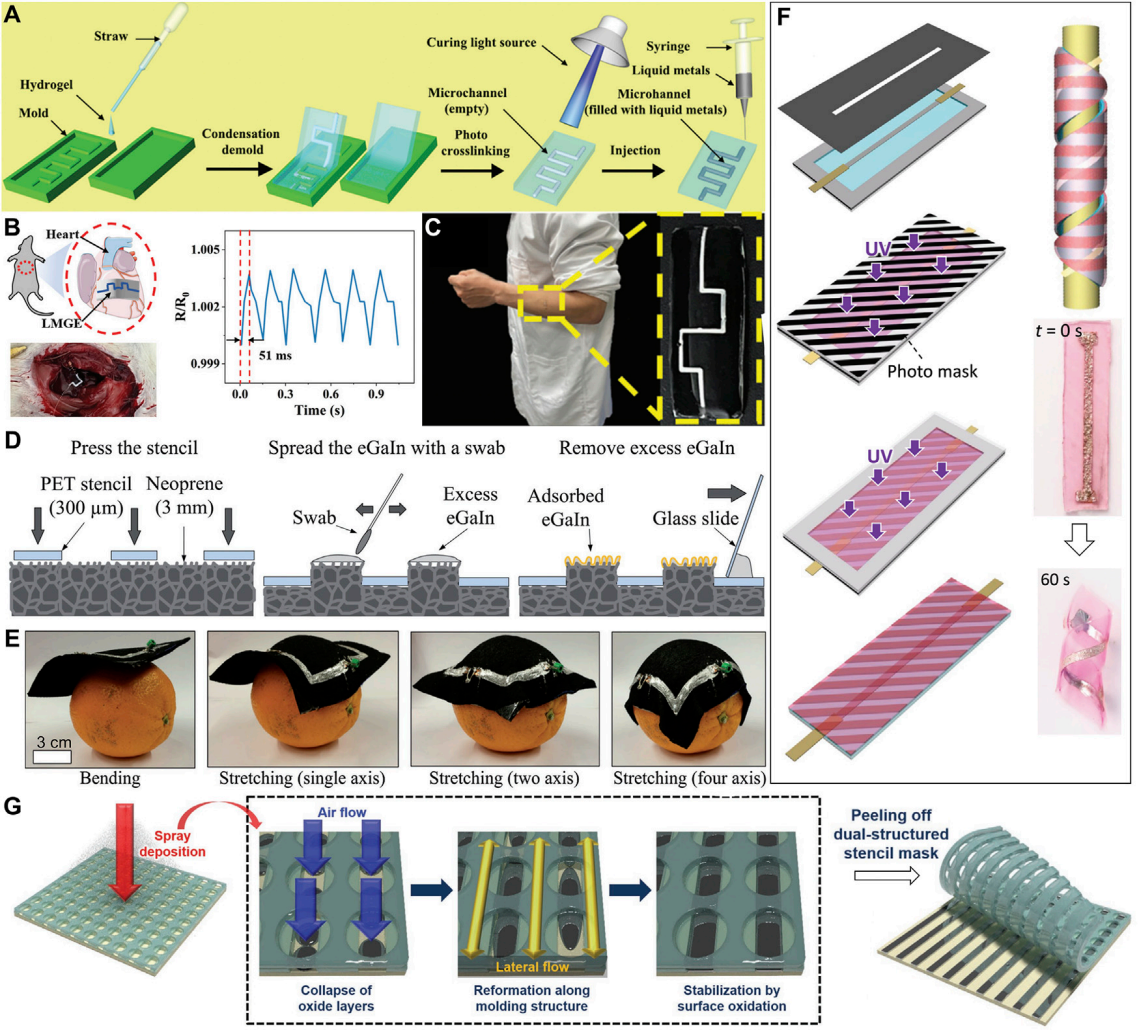
**(A)** The manufacturing process of LMGE (Yuan et al., 2022). Copyright@2022, RSC **(B)** Schematic diagram of LMGE monitoring heartbeat and resistance changes of LMGE as a function of monitoring heartbeat (Yuan et al., 2022). Copyright@2022, RSC **(C)** Potential applications in physiological signals (Yuan et al., 2022). Copyright@2022, RSC **(D)** A stencil printing process for fabrication of stretchable EGaIn conductors on neoprene (Varga et al., 2017). Copyright@2017, Wiley **(E)** Steps of fitting the stretchable coil onto an orange. (Varga et al., 2017). Copyright@2017, Wiley **(F)** Schematic for the fabrication and photos to show the morphing process (Hao et al., 2021). Copyright@2021, Wiley **(G)** Schematic illustration of one-step spray deposition of LM through the polymeric stencil mask with dual structure (Park et al., 2020b). Copyright@2020, Wiley.

Some studies printed LM on a flexible substrate surface, in order to prevent leakage of LM and avoid the potential side effects of LM on the skin. In recent years, flexible electronic devices based on hydrogels have been widely paid attention. Methacrylate gelatin (GelMA) is a gelatin based hydrogel that has been used in a wide range of biological applications, such as tissue repair and drug delivery. He et al. transformed ordinary brittle GelMA into a highly stretchable hydrogel, and injected LM into the internal microchannels of GelMA hydrogel (GELMA-30) to produce flexible electronics that could be attached to the skin (Yuan et al., 2022), as shown in Figure 4A.With the unique biocompatibility, excellent permeability and great mechanical properties of GelMA-30, and the low toxicity, high conductivity and high rheology of LM, GelMA hydrogel electronics with LM(LMGE) can not only monitor the movement changes and even the heartbeat of rats (Figure 4B), but also be used as a wireless device to monitor the secretion produced during human movement (Figure 4C).Besides, the stencil printing method also can realize printing the LM on the flexible substrate (Varga et al., 2017).For example, Varga et al. covered a 300 μm thick stencil on the neoprene substrate. Then a polyester swab is used to apply LM over the openings of the stencil. The LM is pressured on the neoprene substrate by the hairbrush and is attached to the surface of substrate with the function of oxide film. In the end, the excess material is removed by pressing the stencil and swiping it with a glass slide. The preparation process is shown as Figure 4D. Herein, this LM circuit is made into a pattern of coils for acquiring magnetic field, which emanates from the test subject during an MRI scan. Figure 4E shows that, due to the closer contact between the coils and the stuff, the stretchable coils can be conformally attached to the surface of three-dimensional stuff in order to gain better image efficiency.

In order to achieve the conformal adhesion of flexible substrates on three-dimensional surfaces, numerous deformable flexible substrates have been developed. For example, Park’s team proposes a method based on predeformation patterns and thermoforming processes for the fabrication of three-dimensional stretchable LM flexible electronic devices (Choi et al., 2021). This method uses the highly stretchable thermoplastic elastomer styro-ethylene-butene-styrene block copolymer (SEBS) as the substrate, and EGaIn-CP doped with copper particles as the stretching electrode. Wu et al. use the stencil-printing method to print the LM mixture doped metal nickle particles on the deformable hydrogel (Hao et al., 2021). The deformability of hydrogel substrate is shown in Figure 4F. The gradually varied structure is created by programming the stress distribution inside the hydrogel. The deformation of the hydrogel substrate causes its packaging on stuffs and organs. The S hydrogel with double deformation is prepared by multi-step photopolymerization. The bottom layer is a tough and non-swelling P (AAm-co-MAAc) hydrogel. The top layer is a polyacrylamide (PAAm) hydrogel with a striped pattern. The hydrogel wraps itself around the glass rod after it expands in water due to the swelling mismatch between the different gels. The hydrogel based soft electron (HSE) can be fixed on the rabbit heart due to its self-deformation and good interface contact. An acromion was found in the resistance curve during heart venting, like to tidal waves in a healthy heart.

LM can also be sprayed (Guo et al., 2018c; Li B. M. et al., 2022) or embossed (Bhuyan et al., 2023) on the template to achieve the patterning of LM circuits. For example, Park et al. describe a simple and rapid method for creating LM electrodes with highly precise patterns by one-step spray deposition using a polymer mesh mask with dual structure (Park T. H. et al., 2020). The polymer mesh mask used in this study, consists of an upper and lower structure for injection and molding of LM respectively. The polymer mesh mask can be fabricated by soft lithography using a soft PUA mixture. Because the viscous and flexible polymer mesh mask can be contacted conformally with various materials, patterned electrodes can be made by a one-step jet deposition of the mask even on a three-dimensional substrate. The process needs only little seconds without any surface treatment, as is shown in Figure 4G. The upper layer of the mask has perforations for the injection of LM, which serves as a support for the molding pattern. As a result, a variety of complex and tiny patterns, including slender lines and hollow forms, can be easily achieved. It is possible to achieve sub-micron patterns of LM by spraying. The property is shown successfully over the various substrates, including human skin. It shows the potential for the development and further refinement of disposable and reconfigurable wearable electronic devices.

### 4.3 Selectively adhered substrate

**FIGURE 5:**
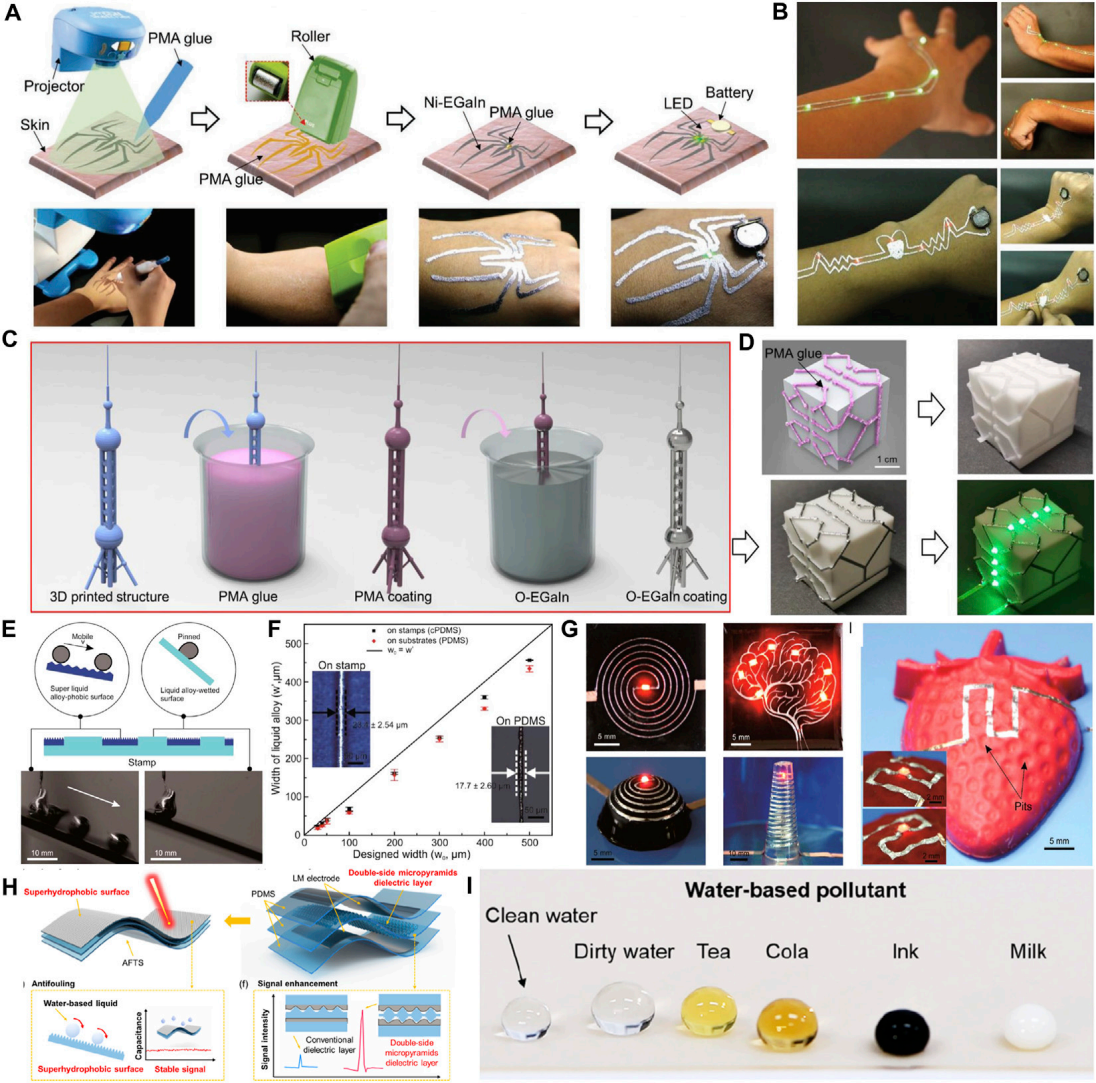
Printing methods based on selective adhesion substrates. **(A)** The schematic diagrams and the photographs of the Ni-EGaIn electronic tattoo printing process (Guo et al., 2019a). Copyright@2019, Wiley **(B)** Two examples of Ni-EGaIn electronic tattoo with LED array (Guo et al., 2019a). Copyright@2019, Wiley **(C)** Brief illustration of the whole process of 3D transfer printing (Guo et al., 2021). Copyright@2021, Elsevier **(D)** The O-EGaIn electronics on the surface of a cube and square pyramid (Guo et al., 2021). Copyright@2021, Elsevier **(E)** A selectively amphiphilic patterned plate for flexography printing of liquid alloy (Zhang et al., 2019). Copyright@2021, ACS **(F)** Designed and measured widths of liquid alloy lines on stamps/plates (UVtreated cPDMS) and receiving/target substrates (PDMS) (Zhang et al., 2019). Copyright@2019, ACS **(G)** These are a helix circuit of ∼200 μm width on various 3D surfaces (Zhang et al., 2019). Copyright@2019, ACS **(H)** Design and principle of the AFTS (Zhang et al., 2022). Copyright@2022, ACS **(I)** Photo of the droplet of water-based pollutant on the superhydrophobic surface of PDMS (Zhang et al., 2022). Copyright@2022, ACS.

Previous studies have found that the human skin has a rough texture structure, which makes it difficult for LM to directly adhere to the skin. Guo et al. developed a method to apply LM directly to the skin based on the difference of adhesion of LM on different substrates (Guo et al., 2019a). Figure 5A shows that the polymer material (PMA) has excellent adhesion to LM, which can firmly adhere to the skin. Besides, the researchers mixed the LM with solid metal particles (Ni) at room temperature to prepare semi-LM materials (Ni-EGaIn) with high viscosity and plasticity. The structure of the electronic tattoo made of this semi-LM material is more stable and can be easily and quickly applied to the skin using a roller. In addition, the study shows a series of feasible applications of LM electronic tattoos, such as arbitrarily large area LED arrays, temperature sensors, human-computer interaction circuits, body surface heating circuits and body surface electrodes (Figure 5B). This kind of LM electronic tattoo is also very convenient to remove by spraying alcohol and wiping it with a paper towel. Without complex equipment, electronic tattoos with any pattern and function can be fabricated, providing a novel solution for personalized medical treatment and wearable devices. Besides, based on the principles above, the LM can be printed to other materials with a rough surface, such as 3D printed structure (Guo et al., 2021). In this study, the researchers first used 3D printing technique to create a series of complex three-dimensional structures, and covered the surface of the three-dimensional structure with polymer coatings with high adhesion to LM materials. After that, the three-dimensional structure is soaked into LM in order to achieve the adhesion of LM to the three-dimensional structure surface, as shown in Figure 5C. Based on the above principle, the polymer coating is covered in a specific area of the three-dimensional structure. The transfer preparation of three-dimensional circuit can be realized by using the selective adhesion of LM on different interfaces, as shown in Figure 5D.

**FIGURE 6:**
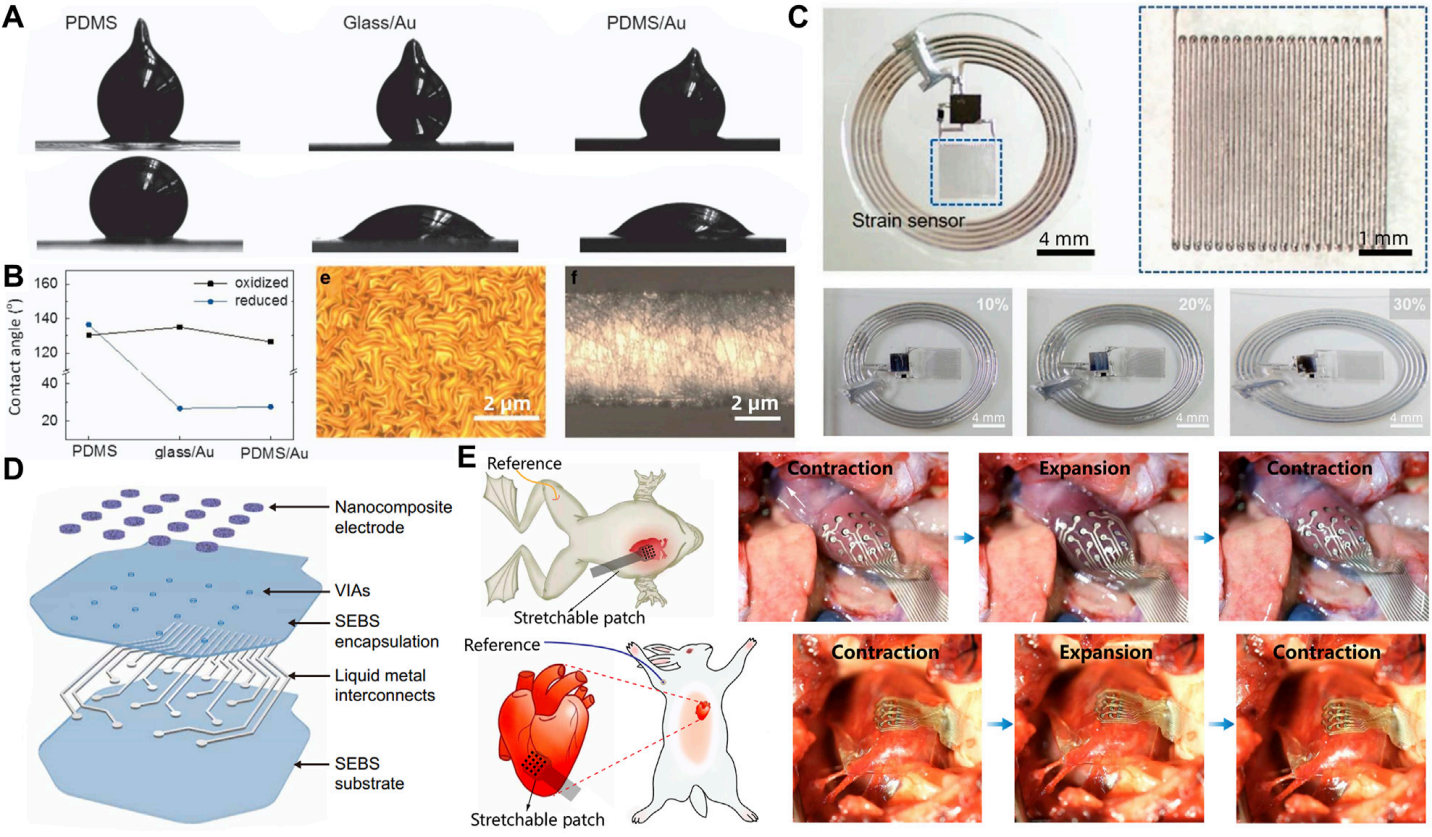
Printing method based on metal welding. **(A)** Contact angle images of the GaInSn droplet on various substrates of PDMS, Au deposited glass substrate and Au deposited PDMS substrate (Jeong et al., 2017). Copyright@2017, Springer nature **(B)** Contact angle measurement of oxidized and reduced GaInSn on various substrates and optical images of Cr/Au film and liquid-metal pattern (Jeong et al., 2017). Copyright@2017, Springer nature **(C)** Optical images of the device under uniaxial strain up to 30% (Jeong et al., 2017). Copyright@2017, Springer nature **(D)** The layer-by-layer construction of the electronic patch (Wang et al., 2022e). Copyright@2022, AAAS **(E)** Schematic illustration of the experimental setup for epicardial electrogram acquisition from a living bullfrog and rabbit (Wang et al., 2022e). Copyright@2022, AAAS.

Similar to the above method, wettability tuning-enabled flexography printing is also a printing technique that makes use of the differences in adhesion of LM on different roughness substrates (Zhang et al., 2019). It was found that the surface of the LM droplet was covered with an oxide film. This oxide film reduces the contact area between the oxide film and the rough substrate, which greatly reduces the adhesion of LM. Using this principle, the smooth surface is treated by laser to increase its roughness, and the part without laser treatment still retains high adhesion to LM. When the treated flexible film is placed on the surface of the LM, the LM can be transferred to a specific position of the flexible substrate to form a LM circuit, as shown in Figure 5E. With this method, the thinnest of LM wires can be up to 20 μm, and the minimum wire spacing can be up to 40 μm, as shown in Figure 5F. This method is simple to operate and convenient to use, and it can also be used for laser treatment of three-dimensional objects to prepare three-dimensional LM electronic circuits (Figure 5G). In addition, Chen et al. use femtosecond laser processing technology to treat the substrate of LM circuit, which can achieve super hydrophobicity of LM circuit (Zhang et al., 2022). As shown in Figure 6H, the femtosecond laser used can achieve programmable, high-efficiency, low-cost and large-scale fabrication of superhard double-sided microcones. Compared with the traditional single-sided micro-pyramid, it greatly improves the pressure sensitivity, LOD and response time due to its enhanced compressibility and reduced viscosity. In addition, the external substrate is treated by femtosecond laser, which gives it excellent antifouling performance and stable inductive signal in a highly humid environment (Figure 5I). Due to the use of fully flexible materials, AFTS can sense external pressure under complex deformation. AFTS has been successfully used to monitor a variety of human physiological and motion signals, showing its potential applications in wearable biological monitoring, man-machine interface and soft robot systems.

### 4.4 Metal welded substrate

Previous studies have proved that LM is easy to infiltrate with solid metal substrate in acidic solution, so coating LM on the surface of rigid metal wire can be used to prevent the electrical properties of rigid metal wire from being broken during stretching (Lim et al., 2022). For example, metal (copper, gold, etc.) lines can be etched in advance on a flexible substrate, and the oxide layer on the metal surface is removed in an acidic solution, so that the LM is in direct contact with the solid metal, resulting in contact wetting (Figure 6A). Causes the LM to attach to the surface of the solid metal line to form a LM line (Figure 6B) (Jeong et al., 2017). Figure 6C shows a resistance strain sensor with an inductance coil, which can be electromagnetically coupled with an external primary coil. All electrical interconnections are composed of GaInSn encapsulated in PDMS, thus forming a three-layer structure. The equipment is intrinsically stretchable at the system level and has stable mechanical and electrical properties under external strain. When the device is connected to the skin of human body, real-time data caused by the deformation of the device can be obtained from the strain sensor, and each deformation corresponds to a specific intensity. By installing multiple devices on the knuckles, various finger movements can be distinguished. In addition, this method can also be used to manufacture high-precision implanted electrode arrays, as shown in Figure 6D (Wang S. et al., 2022). The electrode array is completely composed of compliant mechanical components, including SEBS elastomer as substrate/package, LM for interconnection, carbon nanotube nanocomposites for electrodes, and micro-cracked conductive polymer as bioelectronic interface. The electrode array has ultra-high tensile capacity up to 400% tensile strain and excellent durability against repeated deformation. It has long-term stability, conformal attachment to internal organs and low interface impedance under physiological conditions. ECG signals can be collected on the fast beating hearts of bullfrogs and rabbits, as shown in Figure 6E. The advantage of this method is that it can print high-precision circuits. But the steps of etching solid metal circuits make its preparation cost high, so it’s not conducive to be popularized and used on a large scale.

### 4.5 Transfer printing substrate

**FIGURE 7:**
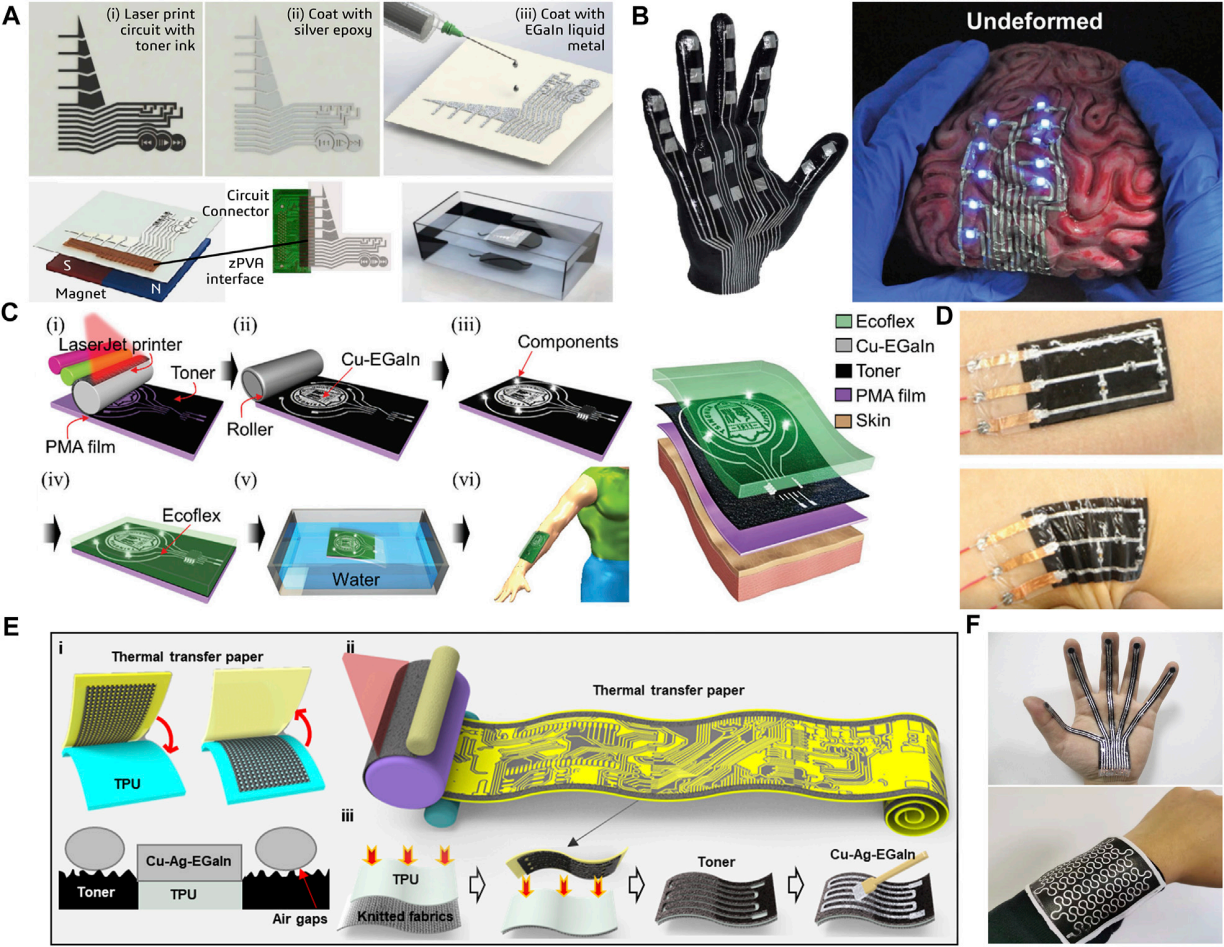
Fast printing method based on transfer printing substrate. **(A)** Fabrication steps including printing, interfacing with microelectronic chips or external circuits, and hydroprinting (Lopes et al., 2018). Copyright@2018, ACS **(B)** Example printed on a 3D printed hand model (Lopes et al., 2018); Copyright@2018, ACS Functioning LEDs on a soft brain-shaped toy (Tavakoli et al., 2018). Copyright@2018, Wiley **(C)** Fabrication of stretchable e-skin and structural diagram of the Cu–EGaIn enabled e-skin (Guo et al., 2020). Copyright@2020, RSC **(D)**. The temperature monitoring circuit transferred to human skin (Guo et al., 2020). Copyright@2020, RSC **(E)** Principle and the fabrication process of the liquid meal conductive traces. (Guo et al., 2022). Copyright@2022, ACS **(F)** LM patterns on smooth substrate and rough substrate (Guo et al., 2022). Copyright@2022, ACS.

In order to realize the patterning of the LM circuits, all the above methods need to customize the template in advance, which increases the cost and time of circuit manufacturing. And it is inconvenient to realize the personalized customization of the LM circuits. In addition, other preparation methods require expensive processing equipment, such as lasers for rough substrates and lithography machines for metal layers, which also increase the manufacturing cost of LM circuits. In order to solve this problem, there have been some methods of using low-cost desktop printing devices to make printing templates. The use of desktop printing equipment greatly reduces the manufacturing cost and time of the template, and can realize the large area printing of LM circuits. For example, Majidi et al. take advantage of the wettability of LM and silver nanoparticles. They used inkjet printers to deposit AgNP ink on the polymer film, dropped it into EGaIn, and rubbed the above products on the circuit with lint-free cloth to cover the entire circuit. Finally, the excess EGaIn is removed with a weak aqueous solution of acetic acid, and the LM circuit is packaged (Tavakoli et al., 2018). The rapid manufacturing of LM electronic tattoos can be realized by combining the above methods with water transfer technology (Lopes et al., 2018). As shown in Figure 7A, immerse it completely in the bath or tank. The “Ag-In-Ga” circuit will then be suspended on the surface of the bath. After a period of time, the water-soluble interlayer of the TTP substrate dissolves and the 5 μm carrier membrane is separated from the substrate paper. The carrier film is attached to the object and matches its surface, and then the object is lifted out of the bath. The electronic tattoos can be conformally applied to complex three-dimensional surfaces, such as 3D-printed prosthetic hand shells, which including LED and capacitive tactile inputs, and brain models with rugged surfaces (Figure 7B).

Compared with the above method of coating silver paste on carbon powder to realize LM adhesion, which the silver paste used adds to the cost of making the circuit, Guo et al. found that toner printed by desktop laser printer has rough surface and poor adhesion to LM (Guo et al., 2019b). The thermal transfer paper coated with PU glue is put into the laser printer. Secondly the pre-designed pattern is printed on the thermal transfer paper coated with PU glue. Then the Ni-EGaIn is printed on the paper with toner pattern by drum. Due to the significant difference in adhesion, Ni-EGaIn is selectively printed on PU adhesive with strong adhesion, instead of the surface of carbon powder. Similarly, by combining this method with water transfer technology, the rapid manufacture of LM electronic tattoos can also be realized (Guo et al., 2020), as shown in Figure 7C. This kind of electronic skin can be used as stretchable electronic products, such as interactive devices, wearable sensors and flexible displays (Figure 7D).In addition, some studies have found that the specially treated toner has high adhesion to the LM, so the LM can be coated on the toner surface and then transferred to other flexible substrate surfaces (Zhao et al., 2020). In addition to the water transfer method, the toner pattern can also be transferred to a flexible substrate to make a template with selective adhesion to LM at a certain pressure and temperature (Guo et al., 2022). As shown in Figure 7E, first use a laser printer to print the toner pattern on the thermal transfer paper. Then, heat the heat transfer printing to 150°C through the heating plate and keep heating for 30 s. At this time, the toner is completely transferred to the substrate film. Apply the prepared Cu-Ag-EGaIn to the basement membrane with a brush, and the Cu-Ag-EGaIn will selectively adhere to the area where there is no toner. On smooth substrates such as thermoplastic polyurethane (TPU), PDMS, polyethylene terephthalate (PET), glass, etc., toner can be directly transferred to the substrate, while on rough substrates such as textile, paper, wood, fruit skin, a layer of TPU film can be hot-pressed on the substrate surface, and then toner transfer printing can be carried out, as shown in Figure 7F.

### 4.6 LM-elastomer composites

**FIGURE 8:**
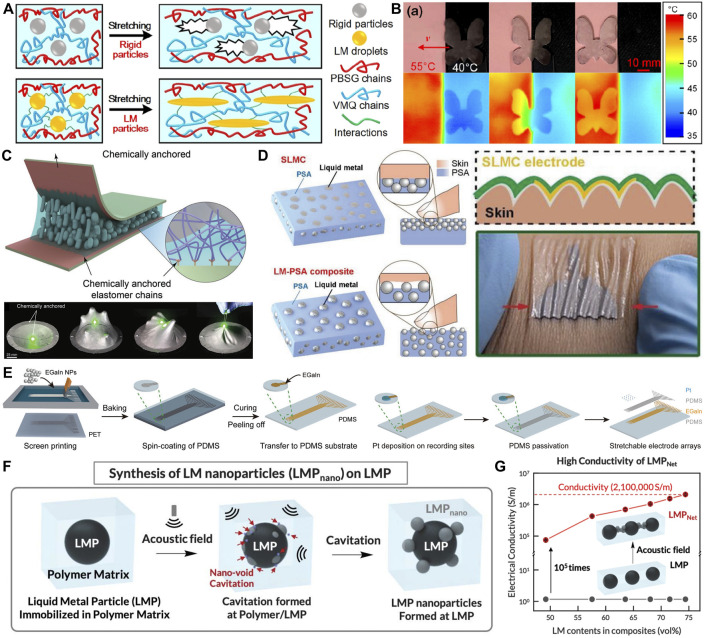
Printing method based on LM micro-nano-particle composite. **(A)** Illustration between the LM/PBSE elastomer and rigid-particle reinforced composites during the tensile process (Zhao et al., 2022). Copyright@2022, Wiley **(B)** The thermal camouflage ability of the LM/PBSE butterfly (Zhao et al., 2022). Copyright@2022, Wiley **(C)** Schematic representation of LM composite adhesion with chemical anchoring. (Pozarycki et al., 2022). Copyright@2022, Wiley **(D)** Schematic illustrations of the structure of the SLMC and the LM-PSA composites. (Tang et al., 2022a) Copyright@2022, Wiley **(E)** Schematic illustration of the fabrication of SEA using screen printing (Dong et al., 2021). Copyright@2021, Wiley **(F)** Schematic of the formation of LM nanoparticles (LMPnano) at the surface of existing micrometer-sized LM particles (LMPs) via acoustic field application (Lee et al., 2022b). Copyright@2022, AAAS **(G)** Electrical conductivity of the LMP–polymer composite as a function of the LM content before and after the LMPNet formation (Lee et al., 2022b). Copyright@2022, AAAS.

The printing technology of this LM micro-nano particle complex is through mixing LM with silica gel and other polymer elastic materials to form tiny LM droplets in silica gel, as shown in Figure 8A (Pei et al., 2022; Zhao et al., 2022).The LM droplets mixed in the polymer can significantly improve the thermal conductivity of the composite (Figure 8B).Because the LM droplets in silica gel are separated from each other, in order to make the LM droplets form a conductive path, it is necessary to use mechanical external force to destroy the droplets, so that the separated LM droplets communicate with each other, and finally form a conductive path (Lee G. H. et al., 2022; Li X. et al., 2022; Kim et al., 2022; Pozarycki et al., 2022).Since LM droplets can be deformed by silica gel stretching, the electronic circuit formed by this LM silica gel compound has high tensile property, as shown in Figure 8C. However, the preparation of the LM silica gel complex requires the mixture of LM in the silica gel material, so it is difficult to recycle the LM, and the circuit function is only the part of the mechanical stress damage, resulting in a large amount of LM waste. Xing Li et al. developed a kirigami-structured LM paper (KLP) (Li X. et al., 2022). KLP can effectively solve the waste problem. Firstly, cellulose nanofibrils (CNFs) are dissolved in deionized (DI) water to form a CNF aqueous solution. Then EGaIn is added to it. After that, poly (vinyl alcohol) (PVA)-water solution was blended with the former solution. Finally, pour the solution into a mold. After being dried, remove the mold and make it a LM paper by kirigami technique. As the experiments show, KLP not only has great conductivity and flexibility, but also the followings. Direct exposure of conductors to skin can improve the accuracy of electrical signal acquisition. LMP can be reprepared by adding LMP to deionized water after shearing. There is no significant change in electrical conductivity and tensile properties. So KLP achieves recyclable feature and environmental protection.

In addition, considering the high adhesion and biosafety of hydrogel materials with human skin (Xu Y. et al., 2021; Yamagishi et al., 2021), adhesive circuits made by mixing LM in hydrogel materials have been widely used. For example, polyethylene glycol (PEG) mixed with PDMS is used as a base adhesive (PPA) to encapsulate gallium-based LM alloy circuits as skin electronic devices (Cheng et al., 2022).As shown in Figure 8D, an intrinsically viscous LM conductor (SLMC) is made by mixing a LM alloy and a pressure-sensitive adhesive (Tang L. et al., 2022).LM is enriched on the surface to form a circuit structure to achieve electronic connection and PSA is used to adhere to the surface of different substances to achieve mechanical connection. It will not be affected by the drying of the hydrogel by air, and will maintain high conductivity and adhesion for a long time, and will not be detached due to excessive deformation of the skin.

In order to avoid the waste of redundant LM composites, traditional screen printing methods can be used to achieve large-scale patterning of LM composites (Ding et al., 2022). Figure 8E shows a manufacturing strategy of highly stretchable neural electrode array based on screen printed LM conductor onto PDMS matrix (Dong et al., 2021).First, the LM composite is screen printed on the PET substrate, and a layer of PDMS is poured on it. After the PDMS is cured, it is stripped from the PET substrate. Then the LM droplets in PDMS are connected through stress stretching. Finally, the Pt coating is covered on the electrode site, and the circuit is packaged with PDMS. In order to realize the conduction of LM droplets in a variety of flexible materials, a LM particle network (LMPNet) was developed (Lee W. et al., 2022).It is assembled by applying a sound field to a solid insulated LM particle composite as an elastic conductor (Figure 8F).The external sound field can make the LM droplets disperse into nanometer droplets and connect with the surrounding LM droplets, thus realizing the conduction of the LM (Figure 8G). LMPNet conductors can produce multiple layers of high-density E-PCB, in which many electronic components are tightly integrated to create highly stretchable skin electronics. In addition, LMPNet can be formed in a variety of polymer substrates, including hydrogels, self-healing elastomers and photoresist, thus showing their potential applications in the field of soft electronics. In summary, this LM composite can effectively prevent the leakage of LM, and can achieve a wide range of tensile deformation. However, the contact area between droplets is limited, and its electrical conductivity is obviously weaker than that of bulk LM.

## 5 Various functional conformal electronics

### 5.1 Multilayer conformal electronics

**FIGURE 9:**
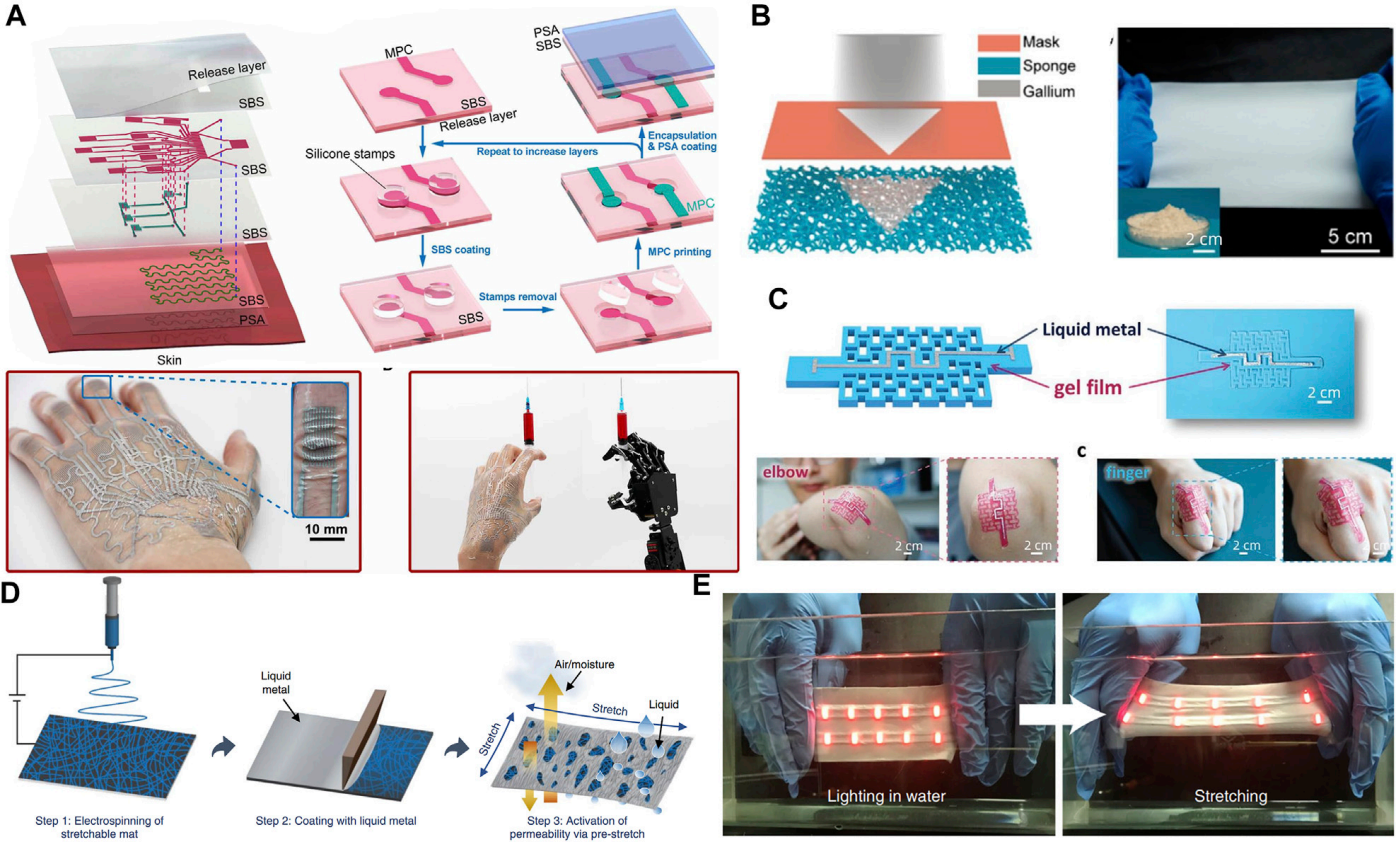
Multilayer and breathable conformal electronics. **(A)** Exploded schematics of the METT containing three circuit layers. (Tang et al., 2021). Copyright@2021, AAAS **(B)** Schematic illustration of the LM micromesh by physical deposition of LM on the metalized sponge with copper. (Li et al., 2022c). Copyright@2022, ACS **(C)** Schematic for the design and the photo of soft electronics. (Yu et al., 2021) Copyright@2021, Wiley **(D)** Schematic illustration of a typical fabrication process of an LMFM (Ma et al., 2021). Copyright@2021, Springer nature **(E)** Images of the encapsulated stretchable mat printed with EGaIn interconnects and mounted LEDs underwater. (Ma et al., 2021). Copyright@2021, Springer nature.

Jiang et al. developed multi-layer electronic tattoos that are thin and highly adhesive (Tang et al., 2021). As shown in Figure 9A, the multi-layer electronic tattoo consists of a bonding layer, a release layer and a circuit module between them. The circuit module of the three-layer METT consists of three layers: the first layer consists of 11 strain sensors, the second layer consists of four strain sensors, and the third layer consists of a heater. Each layer can be prepared by spin coating, and electronic tattoos with any number of layers and thicknesses can be assembled by layer-by-layer preparation. Different layers can be interconnected by holes preset on SBS substrate. This electronic tattoo can be closely attached to the folds of the skin, and can be used to detect the movement of human fingers, and has a broad application prospect in the fields of human-computer interaction.

### 5.2 Breathable conformal electronics

Air permeability is an important performance of conformal electronics applied on the skin to realize the gas exchange between the skin and the surrounding environment, especially the evaporation of sweat under sweating. Using breathable films as the substrate of conformal electronic devices is a relatively easy strategy to consider for achieving the permeability of conformal electronic devices (Li Y. et al., 2022). For example, on the thin copper adhesive layer, SIS elastomer sponge is used as the stretchable substrate, then gallium layer is deposited in the thermal evaporator. Next, using the SIS sponge as a porous template to produce a mesh coating, as shown in Figure 9B. The porous micro mesh shows textile level permeability to achieve long-term wear comfort, and has high tensile (400% strain) and excellent durability. Furthermore, porous structures can also be made on the impermeable hydrogel substrate to achieve the permeability of the substrate (Yu et al., 2021). The hydrogel film with paper-cut structure shown in Figure 9C is prepared by free radical polymerization using a photomask. Ni doped LM alloy was coated on the surface of the film as a hydrogel sensor. The paper-cut structure of the hydrogel film can not only provide air permeability, but also make the hydrogel film have additional extensibility and tight wrapping, which can better fit the skin surface with complex surface structures such as human wrists, elbows, and finger joints. In recent years, the non-woven fabric made of electrospinning has good air permeability due to its porous structure on the surface, which has attracted people’s attention and is widely used to manufacture flexible electronic devices with air permeability (Cao et al., 2022). Similarly, conformal electronic devices with permeability can also be made by combining LM with electrospun films (Zhuang et al., 2021). For example, zheng et al. developed a super elastic, high permeability, biocompatible LM fiber felt (Ma et al., 2021). Figure 9D shows the preparation process of the LM fiber felt. First, the fiber felt is prepared by electrospinning SBS fiber, then coated or printed with LM. Finally, the permeability is activated by pre stretching. LM fiber felt has high permeability, extensibility, conductivity and electrical stability. It also shows good biocompatibility and intelligent adaptability to 1800% strain in all directions. In addition, the circuit can be packaged by electrospinning on the surface of the LM circuit and covering it with a layer of SBS fiber film, which has a certain waterproof property, as shown in Figure 9E.

### 5.3 Degradable conformal electronics

**FIGURE 10:**
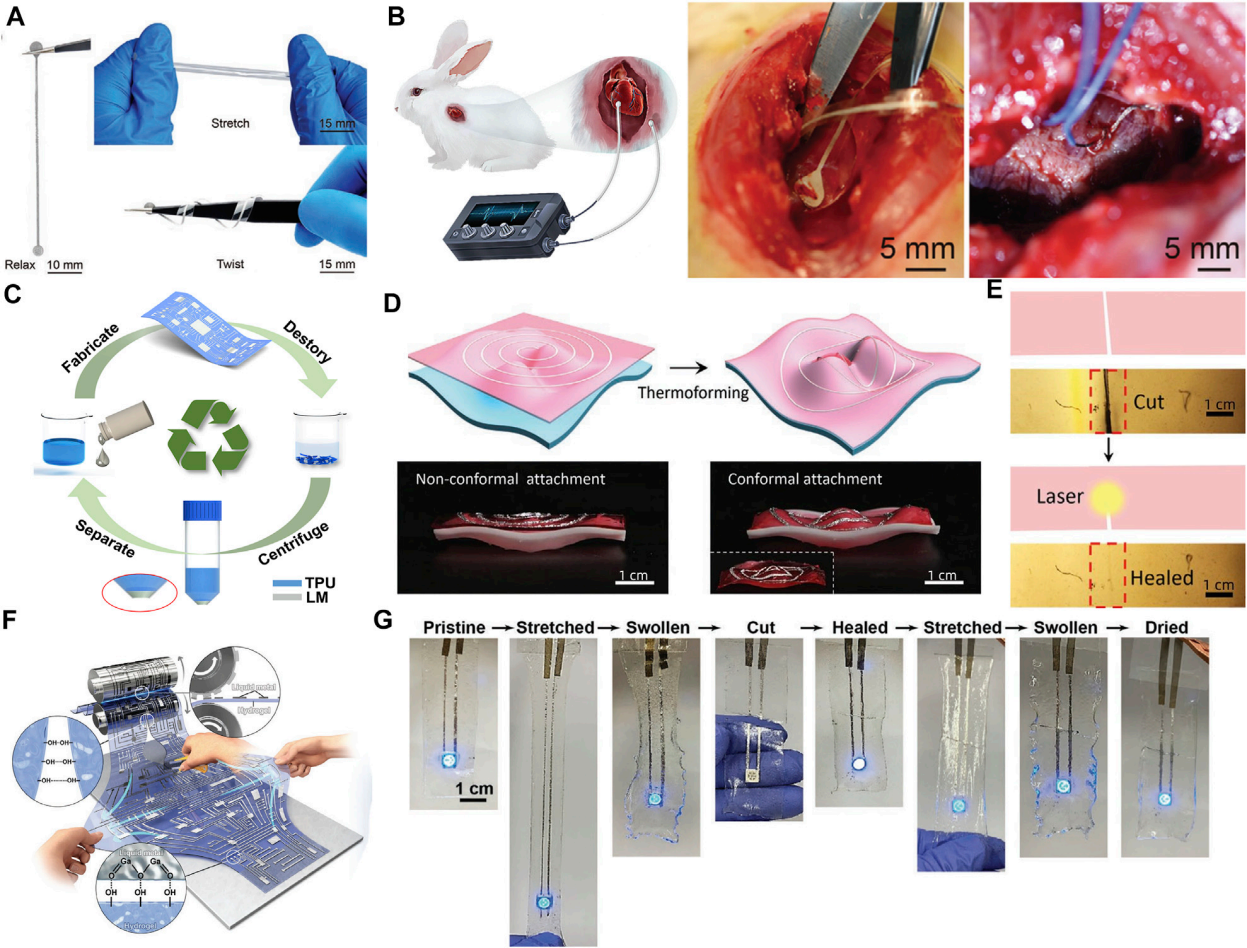
Degradable and self healing conformal electronics. **(A)** Snapshots of the saTPW undergoing different treatments such as relax, stretch, and twist (Hang et al., 2021). Copyright@2021, Wiley **(B)** Illustration of saTPW for correcting abnormal heart rates. (Hang et al., 2021). Copyright@2021, Wiley **(C)** Schematic of the cyclic process of flexible electronic device preparation, recycling, and reconfiguration (Wang et al., 2021b). Copyright@2021, ACS **(D)** Schematic and digital photos of thermoforming process of the hydrogel to devise conformal HSE to set on curved surface (Hao et al., 2022). Copyright@2022, Wiley **(E)** Schematic and photos of laser-induced healing of GA-20-1.5-32 hydrogel (Hao et al., 2022). Copyright@2022, Wiley **(F)** Schematic illustration of a deformable and self-healable EGaIn electrode on a hydrogel (Park et al., 2020a). Copyright@2020, Wiley **(G)** Photographs of an EGaIn electrode on hydrogel under consecutive mechanical conditions of stretching, swelling in water, cutting, and self-healing (Park et al., 2020a). Copyright@2020, Wiley.

Jiang et al. developed a soft and absorbable temporary epicardial pacing wire (saTPW), which can effectively correct abnormal heart rate in rabbit models, such as bradycardia and ventricular premature beats (Hang et al., 2021). During the 2 months of subcutaneous implantation of the rat model, saTPW showed excellent conductivity, flexibility, circulatory stability and little inflammatory reaction. Figure 10A shows that this degradable epicardial pacing line has good mechanical properties and can be stretched and bent. It consists of degradable poly (l-lactic acid co- ε- Caprolactone) (PLC) and LM can be degraded by about 10% (mass loss) in phosphate buffered saline (PBS) after 8 weeks, and by about 13% (mass loss) after 2 months of subcutaneous implantation in rats. Compared with ordinary TPW (tpw20, Ethicon), saTPW can better match with soft tissue, and has less inflammation *in vivo*. It can effectively correct the abnormal heart rate in the rabbit model, as shown in Figure 10B. Moreover, the degradation of LM circuit can also be used for material recycling, realizing the recycling of LM and reducing resource waste. For example, the TPU electrospun film can be decomposed through the mixed solution of DMF and THF (Wang M. et al., 2021). Therefore, the LM circuit printed on the TPU electrospun film is placed in the mixed solution to separate the TPU from the LM, and the LM can be recycled, as shown in Figure 10C.

### 5.4 Self healing conformal electronics

Hydrogel materials can not only provide good interfacial adhesion, but also have good self-healing ability (Wang et al., 2023). Therefore, conformal electronic devices with self-healing function can be fabricated by printing LM on the hydrogel substrate. For example, Figure 10D shows a HSE (Hao et al., 2022), which is a conformal electronic device obtained by printing LM on the surface of gel alginate mixed hydrogel. Based on the temperature mediated sol gel transformation of the hydrogel substrate and the high deformability of LM, the thermal forming ability of HSE can be realized, and two-dimensional planar circuits can be wrapped on three-dimensional surfaces with non-Gaussian curvature. In addition, the local heating of the damaged area of the hydrogel can realize the sol-gel transformation of the hydrogel substrate and achieve the effect of self-healing. Figure 10E shows that the laser is used as the heat source to heat the damaged area, change the fluidity of hydrogel and LM, and make the damaged HES self repair to restore the original state and function. Besides, the self healing function can also be achieved by using the chemical crosslinking agent rich in hydrogel itself without additional temperature control (Park J. E. et al., 2020). The LM alloy hydrogel complex shown in Figure 10F has the advantages of high conductivity, low vapor pressure, low viscosity, high deformation, self-healing, etc. Hydrogels can achieve a wide range of mechanical deformation. The interaction of its hydroxyl group with water molecules and the oxide film of EGaIn makes EGaIn easily diffuse on the hydrogel. In addition, the hydroxyl rich in the hydrogel provides hydrogen bond interaction, which can maintain the electrical performance of the circuit after continuous stretching, water expansion, cuts and self-healing, as shown in Figure 10G.

### 5.5 Connecting line with rigid components

**FIGURE 11:**
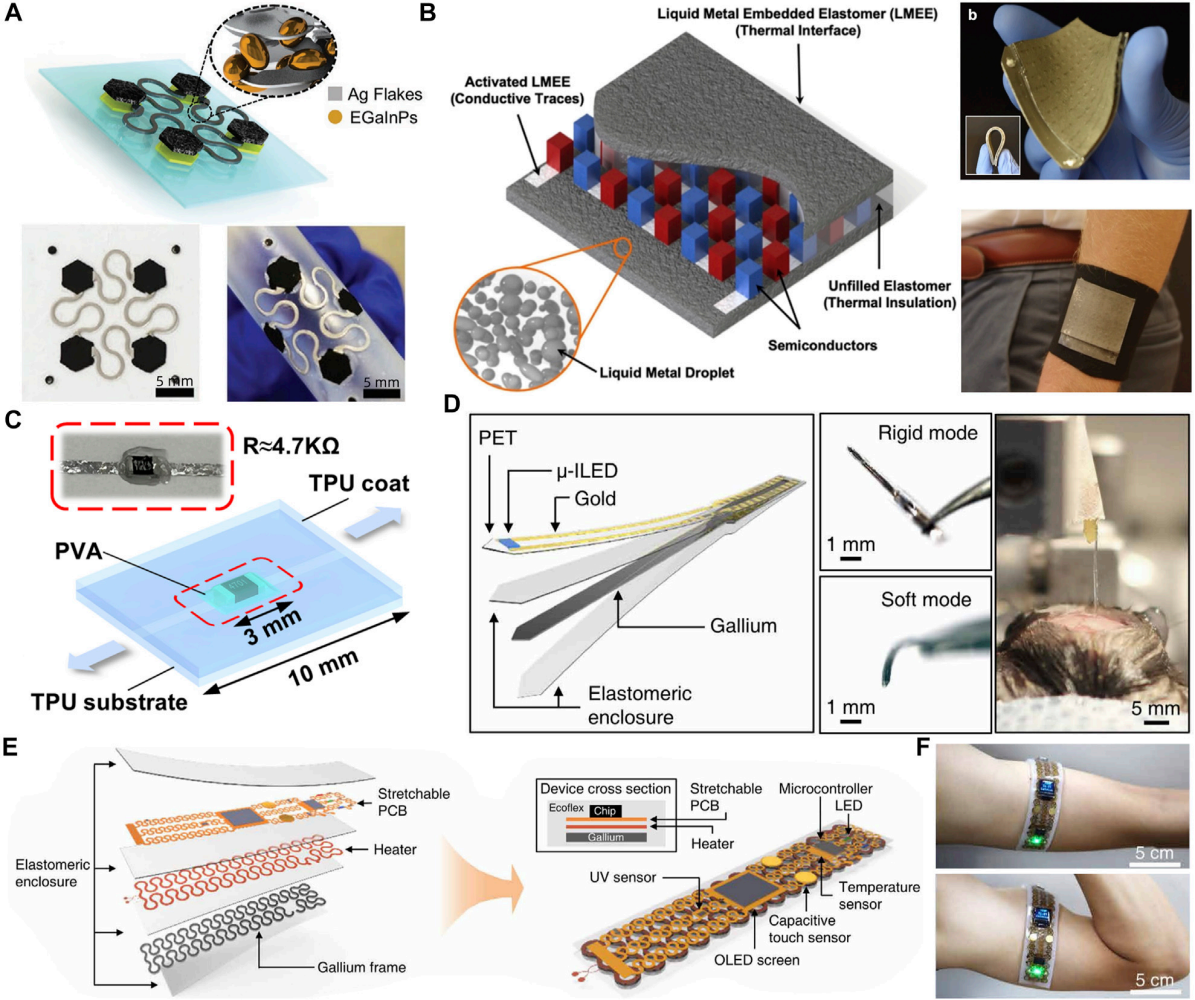
Connecting line with rigid components and flexible circuit with adjustable stiffness. **(A)** Layer-by-layer structural illustration of the all-printed Ag-LM-IB structures. (Silva et al., 2020). Copyright@2020, Wiley **(B)** A schematic of the stretchable TEG with a thermally conductive LMEE as the material interface on the top and bottom. (Zadan et al., 2020). Copyright@2020, ACS **(C)** Schematic of a simplified stretchable electronic device (Wang et al., 2022d). Copyright@2022, ACS **(D)** Conceptual illustration of the fabrication scheme (left) and photographs (right) of the TES neural probe in rigid and soft modes. (Byun et al., 2019). Copyright@2019, AAAS **(E)** A transformative platform for accelerated phase transition from a rigid to soft state. (Byun et al., 2019) Copyright@2019, AAAS **(F)** The sensor on the contracted arm stretched 15% because of muscle volume expansion. (Byun et al., 2019). Copyright@2019, AAAS.

Most functional electronic components are made of rigid silicon based materials, which cannot realize flexibility. Therefore, island bridge structure can be used to divide functional electrodes into several independent rigid “island” compartments and connect them through highly stretchable snake bridge interconnection (Zhao et al., 2021). Figure 11A shows an “island bridge” based on thick film printing process to achieve high stretch (Silva et al., 2020). The printing “bridge” is composed of Ag -LM composite materials with good insulation and inherent extensibility, which is serpentine and is used for high strain deformation to ensure stable interconnection between functional electrodes during large deformation. On the other hand, the printed “islands” are protected by inelastic skeletons, which can withstand high tensile strength and will not deform during stretching. Finally, the strain caused by external deformation can be effectively dispersed around the electrode “island” and contained by the interconnected Ag-LM based “bridge”. In addition, LM can also be used to manufacture wearable energy devices, such as wearable thermoelectric devices (Han et al., 2022; Wei et al., 2022), as the connecting wire of rigid devices. For example, the tensile hot spot generator (TEG) shown in Figure 11B is composed of conductive and thermal conductive LM embedded elastomer (LMEE) composites with n-type and p-type Bi_2_Te_3_ semiconductor integrated arrays (Zadan et al., 2020). The top and top are LMEE layers, which are used as heat exchangers and form electrical connections with Bi_2_Te_3_. LMEE layer is formed by LM dispersed on soft silicon elastomer substrate. The flexible substrate has high extensibility, so that when TEG generates 50% strain, electrical or mechanical failure will not occur. The material used in the thermoelectric generator is well adapted to the texture structure of the skin, and can closely fit when the skin is deformed, with good heat absorption effect. It is worth noting that the reliability of the connection between the rigid inorganic electronic components and the stretchable circuit is very important. To solve this problem, a mechanical gradient strategy was developed to manufacture high-performance stretchable electronic devices (Wang M. et al., 2022). As shown in Figure 11C, polyvinyl alcohol glue is used to fix the integrated circuit on the stretchable circuit, which is made by printing LM on the thermoplastic polyurethane nanofiber film. The strategy of integrated circuit (rigid)—polyvinyl alcohol glue (high elastic modulus) - thermoplastic polyurethane nanofiber film (low elastic modulus) - LM (liquid) realizes the strain gradient during device stretching, thus ensuring stability and reliability. To sum up, using LM as the connecting line of rigid devices can provide attractive and scalable manufacturing of large stretchable soft electronic devices for various sensing, energy and display applications based on various reaction function “island” materials, thus expanding the application of LM in wearable electronic devices.

### 5.6 Flexible circuits with adjustable stiffness

The low melting point of LM can be used to realize the stiffness control of LM circuit within the room temperature range (Sun et al., 2022). For example, based on the mechanically adjustable electronic platform produced by pouring metal gallium into the flexible mold, the temperature triggered soft rigid phase transition realizes the transformation of its shape and stiffness according to the required application, and has high adaptability to various applications (Byun et al., 2019). Figure 11D shows a deep cranial nerve probe with adjustable stiffness realized by phase-change gallium, which remains rigid at the operating room temperature (∼23°C), but will soften after deep brain injection to help adapt to the micro motion occurring in the skull during normal movement. In addition, multi-purpose personal electronic devices that convert between rigid electronic devices and stretchable wearable devices are also made, including a stretchable printed circuit board (PCB) with filiform serpentine copper interconnection, temperature sensors, ultraviolet (UV) sensors, organic LED (OLED) screens, capacitive touch sensors, and microcontrollers for multi-function sensing and user interface (Figure 11E). The device can display time and room temperature, and can also be used as a skin mounted sensor to monitor body temperature and environmental UV index for healthcare and health applications (Figure 11F).

## 6 Discussion

In conclusion, this review discusses the recent research on three-dimensional conformal electronics based on LM, including the unique property of LM that is conducive to the development of conformal electronics, the manufacturing methods of various conformal electronics, and the conformal circuits with multiple functions. Among them, the high conductivity and liquid characteristics of LM enables it to be used as conductive ink for conformal circuit manufacturing. In addition, these LM based conductive inks often has good extensibility. Therefore, the flexible circuit manufactured by them can adapt to large deformation of human skin, organs and other tissues, while other types of conductive inks, such as carbon based inks, are difficult to achieve such large extensibility.

However, it should be noted that the conformal circuits made of LM still have some limitations. In order to further promote the development of conformal electronics in LM, this chapter also discusses some urgent problems and future research directions: First, LM, such as gallium and indium, is rare metal with limited reserves. Compared with carbon based conductive materials, LM is non-renewable resource. And its price is higher than those of common metal materials, such as copper, iron, aluminum, etc. Although the conformal circuit applied on the surface of human tissue uses less LM, its use in the manufacturing of large area flexible electronics will still significantly increase the manufacturing cost. Moreover, disposable electronic tattoo is a mainstream application at present, while LM is not suitable for manufacturing disposable conformal electronic devices. Therefore, the development of new low-cost LM ink is an important research. For example, other cheap metal material can be alloyed or mixed with gallium to reduce the cost of LM. In addition, effective recycling and reuse can avoid the waste of LM. Besides, the patterning method of most LM conformal circuits requires more operation steps and expensive equipment to achieve high-precision flexible circuit printing, which increases the circuit manufacturing cost and operation complexity. Therefore, it is a feasible choice to develop integrated LM flexible circuit printing equipment. This kind of printing equipment needs to integrate multiple operation steps together, and the operator can complete the printing of flexible circuit with simple operation. This kind of universal printing equipment can promote the large-scale use of LM conformal circuits. Finally, the connection stability of rigid electronic components with LM circuits and flexible deformable substrates remains a challenge. New packaging materials and structures need to be developed to maintain the normal function of rigid electronic components in the process of substrate and LM circuit deformation. Some rigid electronic components have many tiny pins, so it is necessary to develop high-precision LM circuit printing methods and ensure the stable connection between many pins and LM wires. In the future, the effective solution of the above problems will enable more applications of conformal electronics based on LM, especially wearable medical devices, including biosensors, neural interfaces, heaters and interconnects.
